# Surgical management for periodic fever, aphthous stomatitis, pharyngitis and cervical adenitis (PFAPA) syndrome: a systematic review and meta-analysis

**DOI:** 10.1016/j.jped.2025.03.006

**Published:** 2025-04-16

**Authors:** Luís Fernando Ferreira Cavalcante, Giovanna Costa, Sophia Massafelli Battistuta, Pedro Faria Makabe, Isadora Silva Fanucci Bueno, Bruno Yuamoto, Felipe Endrigo Gonçalves Vilela, Marco Aurélio Palazzi Sáfadi

**Affiliations:** a Faculdade de Ciências Médicas da Santa Casa de São Paulo, São Paulo, SP, Brazil; b Faculdade de Ciências Médicas da Santa Casa de São Paulo, Departamento de Pediatria, São Paulo, SP, Brazil

**Keywords:** Syndrome of periodic fever, Aphthous stomatitis, pharyngitis, and cervical adenitis, Tonsillectomy, Tonsillotomy, Randomized controlled trial, Meta-analysis

## Abstract

**Objective:**

This systematic review and meta-analysis aimed to evaluate the efficacy of surgical management, specifically total and partial tonsillectomy, for Periodic Fever, Aphthous Stomatitis, Pharyngitis, and Cervical Adenitis (PFAPA) syndrome, focusing on symptom resolution and recurrence reduction.

**Data sources:**

A systematic search of MEDLINE, Scopus, and Cochrane databases identified randomized controlled trials (RCTs) comparing surgical to non-surgical management in children with PFAPA. Data extraction and quality assessment adhered to Cochrane guidelines and PRISMA protocols. Risk ratios (RR) and confidence intervals (CI) were calculated using random-effects models.

**Summary of findings:**

After the removal of duplicates, 31 studies were screened and 3 studies were included. Pooled analysis revealed a 72% reduction in persistent symptoms following surgical interventions compared to non-surgical management (RR: 0.28, 95% CI: 0.12–0.68, I² = 22%, p = 0.005).

**Conclusion:**

The findings confirm that surgical management, including total and partial tonsillectomy, is an effective therapeutic option for PFAPA, with substantial benefits in symptom resolution and quality of life improvement. While surgery offers long-term benefits, its risks, and broader immunological implications require careful consideration. The study underscores the necessity for larger, multicenter trials to validate these findings across diverse populations and optimize treatment strategies.

## Introduction

Periodic Fever, Aphthous Stomatitis, Pharyngitis, and Cervical Adenitis (PFAPA) syndrome is an idiopathic autoinflammatory disorder characterized by recurrent episodes of fever accompanied by pharyngitis, aphthous stomatitis, cervical adenitis, and leukocytosis, with an incidence estimated at 2.3 - 2.6/10,000 children under 5 years in certain populations [1,2]. Typically, the syndrome presents in early childhood, with onset often occurring between the ages of 2 and 5 years. Between episodes, patients have completely asymptomatic intervals, with normal growth and development[2, 3, 4]. Despite its self-limiting nature, PFAPA significantly impacts the quality of life for affected children and their families due to the frequent and predictable episodes of fever and associated symptoms. This cyclical burden can disrupt normal activities and impose psychological and logistical stress on families, as documented in studies emphasizing the profound family impact and diminished health-related quality of life (QoL) during episodes [5,6]. The difficulty in distinguishing episodes of PFAPA from bacterial tonsillitis often results in the overuse of antibiotics in young children, increasing the risk of adverse events, disrupting the microbiota, and contributing to antimicrobial resistance within the community [7].

Although the etiology of PFAPA remains poorly understood, emerging evidence suggests a multifactorial pathogenesis, including a genetic predisposition and dysregulation of innate immune responses. Recent findings have linked PFAPA to genetic variants shared with other autoinflammatory conditions, such as Behçet's disease and recurrent aphthous ulcers, providing clues about their potential underlying mechanisms [8, 9, 10]. Inflammatory markers typically normalize between episodes, underscoring the episodic nature of the disease and its differentiation from infectious etiologies [11].

Treatment options for PFAPA include corticosteroids, which are effective in aborting individual episodes but may increase recurrence frequency [1,3,12]. Other pharmacological preventive approaches, such as cimetidine, montelukast and colchicine, have been used to reduce the frequency and severity of the episodes, but results have shown limited, if any, efficacy [12, 13, 14, 15, 16, 17].

Surgical interventions, particularly tonsillectomy, have gained attention for their potential to provide a curative solution. Observational studies as well as randomized controlled trials (RCTs) results suggest that total or partial tonsillectomy can significantly reduce or resolve PFAPA symptoms, with a reported efficacy of up to 90% in some cohorts [18, 19, 20, 21, 22, 23, 24, 25, 26]. However, the risks of surgical complications, including bleeding and postoperative pain, as well as the long-term immunological impact of these surgical interventions must be weighed against the benefits.

A Cochrane review, based on two small randomized controlled trials, concluded that tonsillectomy has shown significant benefits in children with PFAPA syndrome, including immediate and complete symptom resolution and reduced frequency and severity of episodes. However, the evidence was considered of moderate certainty due to limited sample sizes and applicability concerns. According to the authors’ conclusions, parents must balance surgical risks against medication alternatives, considering that PFAPA often resolves spontaneously, and medications can mitigate episode severity [19].

This systematic review and meta-analysis aim to evaluate the efficacy of surgical interventions for PFAPA syndrome, specifically total and partial tonsillectomy, by analyzing randomized controlled trials. The authors seek to determine the impact of surgical treatment on symptom resolution, recurrence rates, and overall disease burden compared to non-surgical management. By consolidating the latest RCT evidence, this study provides a clearer understanding of the therapeutic potential of surgery, aiding clinicians and caregivers in making informed treatment decisions for children with PFAPA.

## Material and methods

The review protocol was registered in the International Prospective Register of Systematic Reviews (PROSPERO) under the protocol CRD42024569470 on DATA. The systematic review and meta-analysis were conducted and reported in accordance with the guidelines outlined in the Cochrane Handbook for Systematic Reviews of Interventions and the Preferred Reporting Items for Systematic Reviews and Meta-Analyses (PRISMA) [27].

### Eligibility criteria

The studies that met all the following eligibility criteria were included in this meta-analysis: (1) population of children with PFAPA at randomization; (2) intervention group with surgical management; (3) control group with non-surgical management; (4) randomized trials. In addition, studies were only included if there was a report of at least one outcome of interest.

### Search strategy and data extraction

The authors systematically searched MEDLINE, Scopus, and Cochrane databases with each respective RCT filter according to The Cochrane Handbook for Systematic Reviews of Interventions until August 2024 with the following search strategy: “PFAPA AND (tonsillectomy OR tonsillotomy OR adenoidectomy)”. Two authors (G.C. and S.B.) independently extracted the data following predefined search criteria and quality assessment.

### Outcomes

The outcome of interest was the proportion of patients without symptoms of PFAPA after surgery or randomization.

### Quality assessment

The authors evaluated the quality of the RCTs using version 2 of the Cochrane risk-of-bias tool for randomized trials (RoB 2). This tool categorizes studies as having a high, low, or unclear risk of bias across five domains: selection, performance, detection, attrition, and reporting biases [28].

### Statistical analysis

To compare effects for binary outcomes, the authors utilized risk ratios (RR) with 95% confidence intervals (CI). Heterogeneity was assessed using I² statistics, with P values less than 0.10 and I² greater than 25% indicating significant heterogeneity. The authors employed the Mantel-Haenszel statistical method and the random-effects analysis model via Review Manager for the outcome of interest.

## Results

### Study selection and characteristics

**Figure 1:**
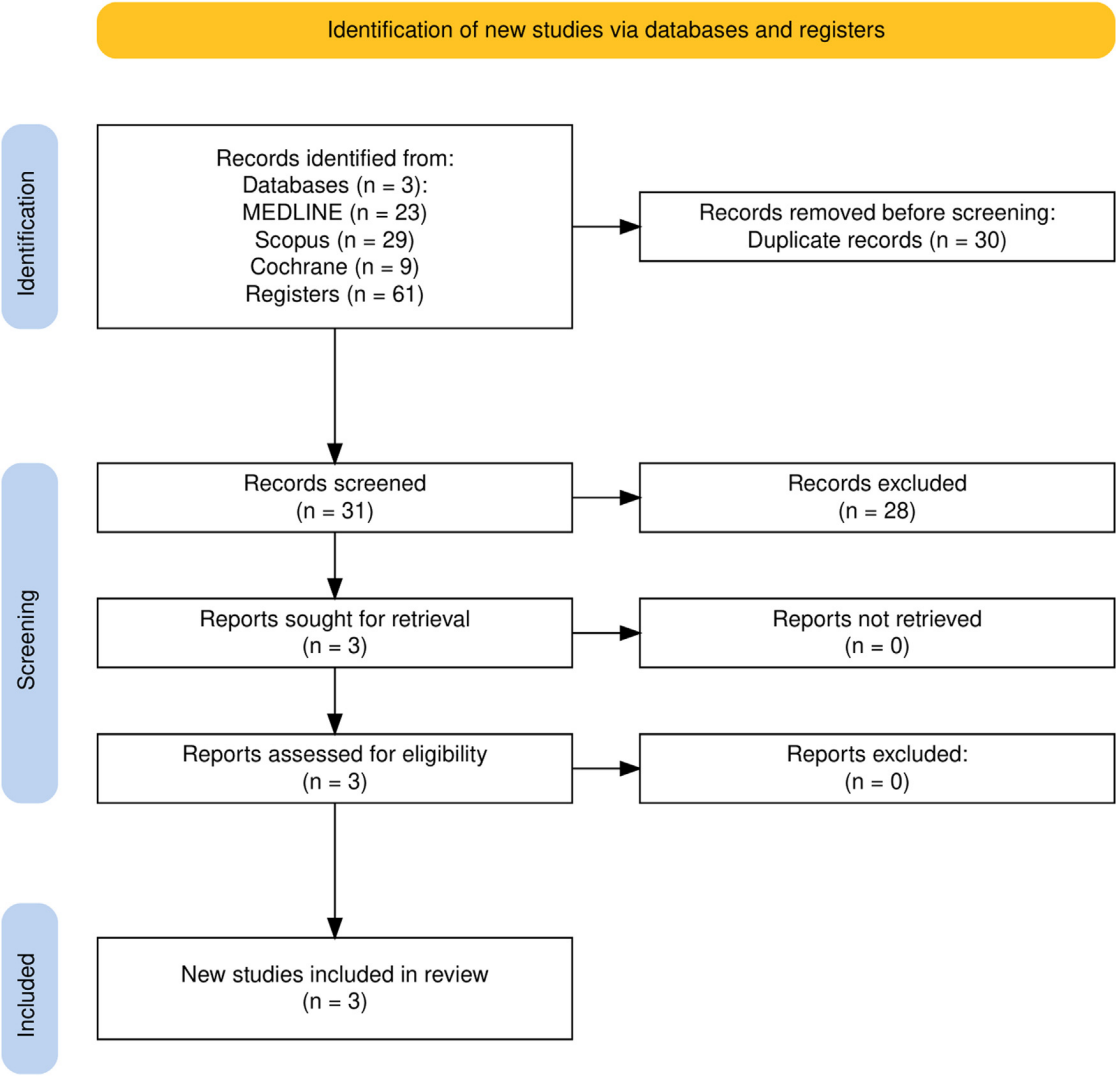
PRISMA flow diagram of study screening and selection.

As detailed in Figure 1, the initial search yielded 61 registers. After the removal of duplicate records, 31 studies were screened. From these, three studies were included. A total of 81 patients were evaluated from 3 RCTs, of whom 41 (50.6%) were in the surgery group, and 40 (49.4%) in the placebo group. Study characteristics are reported in Table 1.

**Table 1:** Baseline characteristics of participants from the included studies.

Study	Intervention	Number of patients		Mean age (years)		Female sex (%)		Mean age at the onset of episodes (years)		Duration of episodes (days)	
		Surgery	No treatment	Surgery	No treatment	Surgery	No treatment	Surgery	No treatment	Surgery	No treatment
**Renko [**24**]**	Tonsillectomy	14	12	4.2	4.0	43	33	NA	NA	3.4	3.8
**Garavello [**25**]**	Tonsillectomy and Adenoidectomy	19	20	5.4	4.9	53	65	2.9	3.1	3.3	3.5
**Lantto [**26**]**	Tonsillotomy	8	8	3.9	4.5	50	25	3.8	3.0	3.5	4.2

NA, not available.

### Pooled analysis of all studies

**Figure 2:**
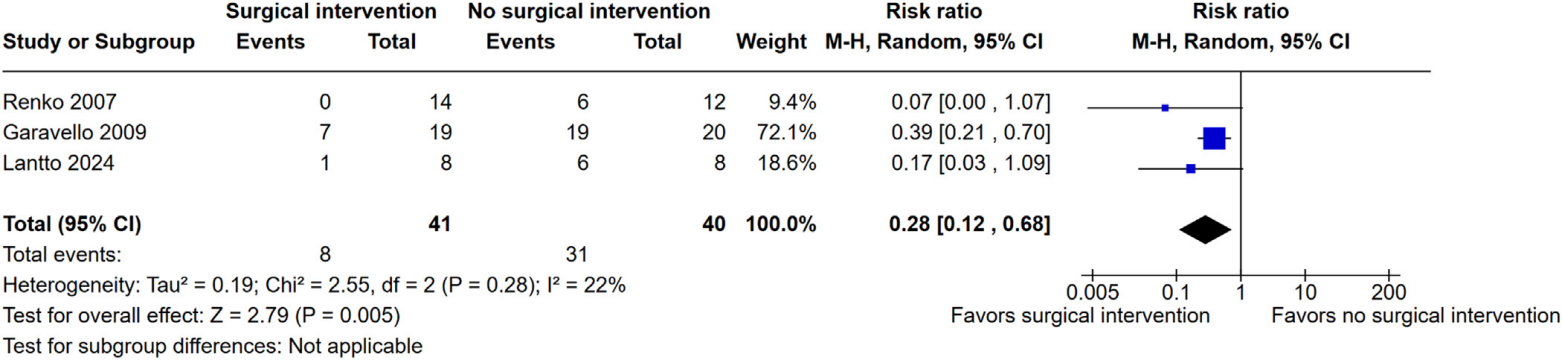
Pooled analysis of the proportion of patients without symptoms of PFAPA after surgery or randomization compared to non-surgical management.

Pooled analysis demonstrated a statistically significant reduction in PFAPA symptom persistence after surgery. The relative risk was 0.28 (95% CI: 0.12-0.68; I² = 22%; p = 0.005), indicating a 72% lower risk of persistent symptoms in the surgical group compared to the control group (Figure 2).

### Quality assessment

**Figure 3:**
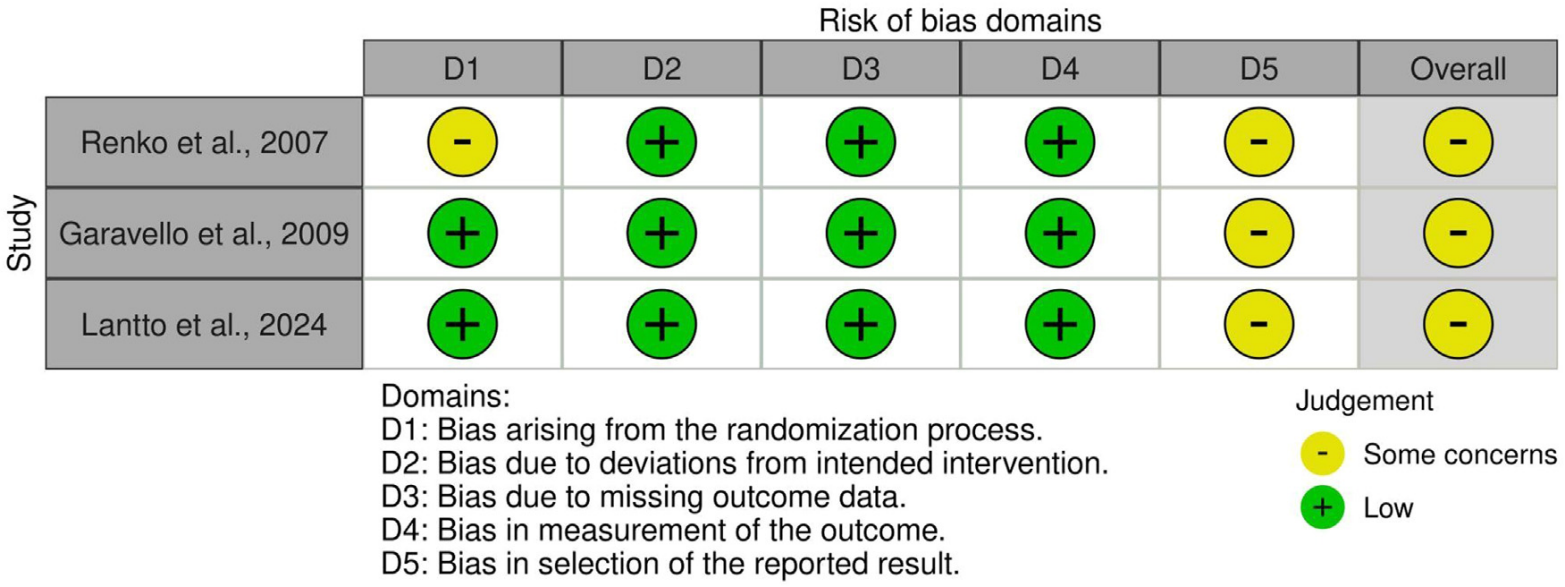
Risk of bias assessment.

Individual RCT appraisal is reported in Figure 3.

## Discussion

This meta-analysis, which included three RCTs involving 81 children with PFAPA, provides compelling evidence for the efficacy of surgical interventions in reducing symptom persistence. The pooled analysis demonstrated a 72% lower risk of persistent symptoms in the surgical group compared to the control group, highlighting the significant therapeutic potential of tonsillectomy and related procedures.

The precise mechanisms underlying the observed efficacy of surgical interventions remain unclear and may be attributed to the removal of a potential nidus of inflammation within the tonsillar tissue, which is hypothesized to play a central role in the pathogenesis of PFAPA [2]. Genetic studies have also suggested that PFAPA is linked to dysregulated immune responses, which could be mitigated by surgical removal of lymphoid tissues [2,29].

Furthermore, the meta-analysis included evidence from recent RCT that investigated the efficacy of newer surgical techniques, such as partial tonsillectomy (tonsillotomy), adding consistency to previous trial results. Although with lower efficacy when compared to total tonsillectomy, tonsillotomy has shown promise in reducing postoperative complications (faster recovery times and lower levels of postoperative pain), making it an attractive alternative for certain patient populations [22,26,30].

Interestingly, partial tonsillectomy may preserve some of the immunological functions of the tonsillar tissue, potentially offering a balance between symptom relief and maintaining partial local immunity. This aspect is particularly relevant in light of findings that total tonsillectomy may increase the risk of viral infections, as observed during the Coronavirus disease 2019 pandemic [31]. This suggests that the immunological role of the tonsils, particularly in pediatric patients, may provide protective barriers against upper respiratory tract infections. Future research is essential to understand the broader immunological consequences and to optimize surgical approaches to minimize adverse effects while maintaining the therapeutic benefits. Ultimately, the choice between total or partial tonsillectomy should be guided by patient-specific factors, including the severity of symptoms, the risk of complications, and the anticipated long-term benefits.

The findings of this meta-analysis, which included only randomized clinical trials, are corroborated by the results of several other observational, retrospective studies that also found these surgical interventions associated with better outcomes and reduced frequency, duration, and intensity of postoperative episodes in PFAPA syndrome [22,23]. In addition to symptom resolution, surgical interventions appear to also significantly improve QoL for both patients and their families. Studies have shown that parents of children undergoing surgical treatment reported reduced stress and improved overall family dynamics [32,33]. These improvements, together with the potential reduction in the inappropriate use of antibiotics during episodes of PFAPA [7], underscore the broader impact of effective management beyond symptom relief.

Although this study primarily focuses on the efficacy of surgical interventions, it is important to consider how tonsillectomy compares to pharmacological treatments such as corticosteroids, colchicine, and cimetidine. Corticosteroids are known to effectively abort individual episodes but may increase recurrence rates. Colchicine and cimetidine have been explored as potential preventive treatments, but their efficacy remains inconsistent [12, 13, 14, 15, 16, 17]. Compared to these medical therapies, tonsillectomy offers a more definitive resolution of symptoms, reducing the frequency of episodes and improving quality of life.

While this meta-analysis demonstrates the efficacy of surgical interventions for PFAPA, several limitations warrant consideration. The small sample sizes and predominantly European focus of the included studies restrict the generalizability of the findings. The small sample size may impact the statistical power of these findings and limit the ability to detect potential differences in treatment outcomes. Regional differences in healthcare access, genetic predispositions, and environmental factors could influence treatment outcomes. Future research should aim to evaluate surgical interventions in larger sample sizes and more diverse populations to determine whether these factors affect the effectiveness of tonsillectomy. Studies conducted in non-European regions could provide valuable insights into potential variations in patient responses to surgery. For instance, the presence of certain MEFV autoinflammatory gene variants has been associated with a higher frequency of surgical failures [34], underscoring the need for geographically diverse trials. Moreover, factors such as age of onset, specific symptom patterns, and coexisting conditions like Familial Mediterranean Fever may influence individual responses to tonsillectomy [35,36]. Despite these limitations, the results presented in this meta-analysis are consistent with other studies in diverse populations, such as the Turkish cohort by Yıldız M et al.,[37] which reported a 95.4% resolution rate of PFAPA cases following tonsillectomy/adenoidectomy. Finally, the limited long-term follow-up data in the included randomized controlled trials restricts our understanding of the durability of surgical benefits and the potential for late complications, such as recurrent infections or scarring [31,33]. Future research should prioritize larger, multi-center trials with diverse populations and extended follow-up periods to address these limitations.

A notable challenge in studying PFAPA is its underdiagnosis due to limited awareness and the absence of specific diagnostic biomarkers. This gap not only hampers accurate prevalence estimates but also restricts the feasibility of large-scale trials. Future research should aim to identify reliable biomarkers and to improve diagnosis and predict treatment response. Elucidating these mechanisms will be of paramount importance to pave the way to optimize surgical or even non-surgical therapeutic innovations.

Lastly, pediatricians and healthcare providers must balance the risks of surgery, including anesthesia-related complications and potential postoperative bleeding, against its benefits. Clear guidelines and shared decision-making with families are crucial to optimizing outcomes and aligning treatment strategies with patient and family priorities.

## Conclusion

This meta-analysis underscores the efficacy of surgical interventions, particularly tonsillectomy, in reducing the persistence of PFAPA symptoms. While the findings support the role of surgery as a viable treatment option, further research is needed to address the limitations of existing studies, including small sample sizes, geographic constraints, and limited long-term data. Future investigations should prioritize larger, multicenter trials and explore the underlying mechanisms of PFAPA to optimize treatment strategies and patient outcomes.

## Funding

This study was not funded.

## Conflicts of interest

The authors declare no conflicts of interest. All authors take responsibility for all aspects of the reliability and freedom from bias of the data presented and their discussed interpretation.
